# Supplementary material for: Rapid detection of inter-clade recombination in SARS-CoV-2 with Bolotie
Source: bioRxiv. 2020 Sep 21:2020.09.21.300913. Preprint. [Version 2] doi: 10.1101/2020.09.21.300913 (PMC7523100; doi:10.1101/2020.09.21.300913)
Supplement: Supplement 1 [file media-1.pdf]

Rapid detection of inter-clade recombination in SARS-CoV-2 with Bolotie

Ales Varabyou [1,2*†], Christopher Pockrandt [1,3*†], Steven L. Salzberg [1,2,3,4], and Mihaela Pertea [1,2,3*]

1 Center for Computational Biology, Johns Hopkins University, Baltimore, MD
2 Department of Computer Science, Johns Hopkins University, Baltimore, MD
3 Department of Biomedical Engineering, Johns Hopkins University, Baltimore, MD
4 Department of Biostatistics, Johns Hopkins University, Baltimore, MD
† These authors contributed equally to this work
*Corresponding author.
Email: ales.varabyou@jhu.edu, pockrandt@jhu.edu, mpertea@jhu.edu

Supplementary Materials

Figures

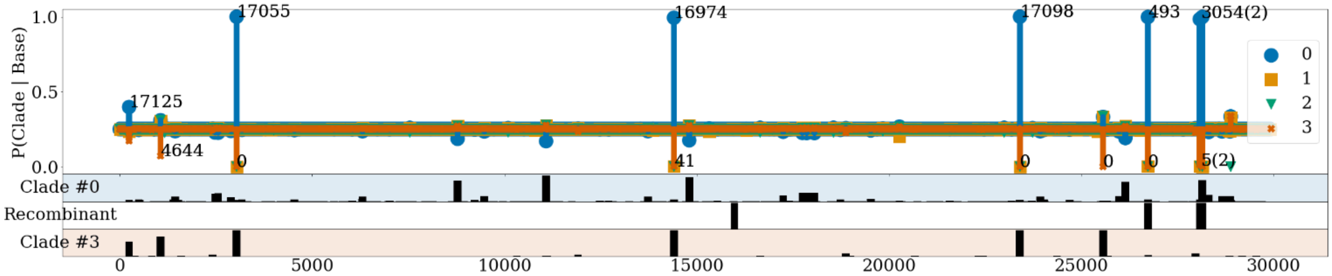

Supplementary Figure 1. *Conditional probability signature of the EPI_ISL_454983 isolate which was not reported as anomalous. The top section shows conditional probabilities of a clade given a nucleotide at each position. Bars are plotted for the two parent clades as reported in* VanInsberghe et al. (VanInsberghe et al., 2020) *and the other clades are shown in dots of corresponding color. Each peak over 0.1 above the baseline (0.25) is labeled with the number of genomes it appears in. An average is reported for multiple variants in close proximity on the plot, listing the number of averaged variants in parentheses. Three bottom sections show the frequency of variants at each position for parental clades (top and bottom rows) and variants observed on the recombinant genome (middle row)*

Tables

**Bolotie**		**GISAID**		**NextStrain**				
Cluster	***# Genomes***	***Clade***	***# Genomes***	***19A***	***19B***	***20A***	***20B***	***20C***
0	1059	L	199	199	0	0	0	0
		S	279	3	276	0	0	0
		V	113	113	0	0	0	0
		O	468	394	13	36	23	2
1	1005	G	1005	9	0	939	50	7
2	1203	GR	1203	0	0	0	1203	0
3	772	GH	772	0	0	334	1	437

Supplementary Table 1. *Mappings between clade identifiers in our analysis and those defined by GISAID* (Shu & McCauley, 2017) *and NextStrain* (Hadfield et al., 2018). *Only 4,494 sequences for which NextStrain assignments were available were counted for this table. Clades in our analysis were taken directly from GISAID, however, smaller lineages L,S,V as well as sequences labeled as "other" (O) were grouped together as sharing neighborhood on the tree.*

**Sequence Identifier**
hCoV-19_Turkey_KOU-MG_2020_EPI_ISL_476832_2020-04-27
hCoV-19_Bahrain_920268866_2020_EPI_ISL_510531_2020-06-23
hCoV-19_SaudiArabia_KAUST-JEDDAH888_2020_EPI_ISL_513037_2020-05-24
hCoV-19_Uganda_UG003_2020_EPI_ISL_451185_2020-03-27
hCoV-19_Bosnia_and_Herzegovina_01-Livno_2020_EPI_ISL_462753_2020-05-27
hCoV-19_Russia_StPetersburg-RII8935S_2020_EPI_ISL_450292_2020-04-21
hCoV-19_Austria_Graz-MUG9_2020_EPI_ISL_437299_2020-04-09
hCoV-19_Austria_Graz-MUG3_2020_EPI_ISL_437199_2020-03-31
hCoV-19_Croatia_297_Varazdin_2020_EPI_ISL_454592_2020-03-05

hCoV-19_Mali_M002673_2020_EPI_ISL_487455_2020-04-10
hCoV-19_Croatia_1761_Dubrovnik_2020_EPI_ISL_454588_2020-03-20
hCoV-19_Czech_Republic_IAB_1_2020_EPI_ISL_426883_2020-03-27
hCoV-19_Oman_RESP-20-4943_2020_EPI_ISL_525425_2020-03-23
hCoV-19_Gambia_0214_2020_EPI_ISL_471158_2020-03-29
hCoV-19_SaudiArabia_KAUST-MADINAH609_2020_EPI_ISL_513172_2020-05-06

Supplementary Table 2. *List of SARS-CoV-2 genomes flagged by Bolotie as recombinant out of 4,494 representative isolates present in the NextStrain tree at the time of the analysis.*

Clade	# Recombinants	# Parents
0	109	171
1	111	41
2	5	148
3	0	90
Total	225	450

Supplementary Table 3. *Summary of the GISAID clade assignment of recombinant genomes and inferred parents.*

Recombinant	Parent #1			Parent #2		
Recombinant	***Clade***	***Segment***	***GISAID***	***Clade***	***Segment***	***GISAID***
EPI_ISL_453713	0	0-4389	EPI_ISL_474909	2	4389-29902	EPI_ISL_457724
EPI_ISL_453712	0	0-4389	EPI_ISL_522572	1	4389-29902	EPI_ISL_442358
EPI_ISL_481145	0	0-4389	EPI_ISL_422772	1	4389-29902	EPI_ISL_458053
EPI_ISL_478027	0	0-14804	EPI_ISL_459691	1	14804-29902	EPI_ISL_477884
EPI_ISL_425846	0	0-4389	EPI_ISL_414593	1	4389-29902	EPI_ISL_469073
EPI_ISL_453753	0	0-4389	EPI_ISL_474909	2	4389-29902	EPI_ISL_420261
EPI_ISL_453752	0	0-4389	EPI_ISL_417203	1	4389-29902	EPI_ISL_442479
EPI_ISL_489588	0	0-8916	EPI_ISL_488600	1	8916-29902	EPI_ISL_428679
EPI_ISL_453714	0	0-4389	EPI_ISL_522572	2	4389-29902	EPI_ISL_418317
EPI_ISL_510531	0	0-4389	EPI_ISL_500635	2	4389-29902	EPI_ISL_496559
EPI_ISL_479572	0	0-28656	EPI_ISL_447565	2	28656-29902	EPI_ISL_465904
EPI_ISL_453729	0	0-4389	EPI_ISL_417203	2	4389-29902	EPI_ISL_462249
EPI_ISL_491989	0	0-4389	EPI_ISL_517362	2	4389-29902	EPI_ISL_435124
EPI_ISL_507986	3	0-27877	EPI_ISL_428790	2	27877-29902	EPI_ISL_421199
EPI_ISL_422850	1	0-20134	EPI_ISL_510174	0	20134-29902	EPI_ISL_455008
EPI_ISL_437472	1	0-20134	EPI_ISL_428687	0	20134-29902	EPI_ISL_433299
EPI_ISL_513037	3	0-27877	EPI_ISL_436478	2	27877-29902	EPI_ISL_443672
EPI_ISL_459371	3	0-27877	EPI_ISL_417336	2	27877-29902	EPI_ISL_443781
EPI_ISL_450292	1	0-14877	EPI_ISL_423026	0	14877-29902	EPI_ISL_462185
EPI_ISL_496321	2	0-20980	EPI_ISL_495677	1	20980-29902	EPI_ISL_443697
EPI_ISL_497594	2	0-20980	EPI_ISL_497589	1	20980-29902	EPI_ISL_439416
EPI_ISL_497455	2	0-20980	EPI_ISL_496222	1	20980-29902	EPI_ISL_447718
EPI_ISL_496334	2	0-21614	EPI_ISL_495706	1	21614-29902	EPI_ISL_426986
EPI_ISL_525424	2	0-18486	EPI_ISL_491128	0	18486-29902	EPI_ISL_416044
EPI_ISL_497021	0	0-14804	EPI_ISL_437300	2	14804-29902	EPI_ISL_423582
EPI_ISL_525428	2	0-18486	EPI_ISL_458116	0	18486-29902	EPI_ISL_416580
EPI_ISL_468407	3	0-18736	EPI_ISL_418037	0	18736-29902	EPI_ISL_426512
EPI_ISL_508098	3	0-27384	EPI_ISL_429316	2	27384-29902	EPI_ISL_517083
EPI_ISL_520686	1	0-20134	EPI_ISL_457985	0	20134-29902	EPI_ISL_483126

EPI_ISL_495767	0	0-28310	EPI_ISL_463692	2	28310-29902	EPI_ISL_481807
EPI_ISL_476832	0	0-14804	EPI_ISL_426680	1	14804-29902	EPI_ISL_417790
EPI_ISL_450122	0	0-4389	EPI_ISL_454919	1	4389-29902	EPI_ISL_460086
EPI_ISL_420268	0	0-4389	EPI_ISL_436738	2	4389-29902	EPI_ISL_420278
EPI_ISL_438422	3	0-27877	EPI_ISL_418425	2	27877-29902	EPI_ISL_436293
EPI_ISL_518315	2	0-28878	EPI_ISL_520048	0	28878-29902	EPI_ISL_479960
EPI_ISL_487348	2	0-28869	EPI_ISL_482867	0	28869-29902	EPI_ISL_427453
EPI_ISL_518193	2	0-28878	EPI_ISL_522218	0	28878-29902	EPI_ISL_518855
EPI_ISL_508009	3	0-27877	EPI_ISL_508873	2	27877-29902	EPI_ISL_523989
EPI_ISL_520527	2	0-28878	EPI_ISL_521787	0	28878-29902	EPI_ISL_499667
EPI_ISL_519690	2	0-28878	EPI_ISL_521589	0	28878-29902	EPI_ISL_479960
EPI_ISL_504177	3	0-27877	EPI_ISL_513259	2	27877-29902	EPI_ISL_469830
EPI_ISL_519112	2	0-28878	EPI_ISL_521787	0	28878-29902	EPI_ISL_499667
EPI_ISL_521605	2	0-28878	EPI_ISL_522114	0	28878-29902	EPI_ISL_479960
EPI_ISL_512969	3	0-27877	EPI_ISL_512953	2	27877-29902	EPI_ISL_480264
EPI_ISL_475026	3	0-27877	EPI_ISL_418430	2	27877-29902	EPI_ISL_454064
EPI_ISL_518953	2	0-28878	EPI_ISL_521585	0	28878-29902	EPI_ISL_499667
EPI_ISL_521775	2	0-28878	EPI_ISL_521796	0	28878-29902	EPI_ISL_479960
EPI_ISL_512983	0	0-3095	EPI_ISL_437716	3	3095-29902	EPI_ISL_512981
EPI_ISL_436055	0	0-3095	EPI_ISL_482575	3	3095-29902	EPI_ISL_420797
EPI_ISL_521286	2	0-28878	EPI_ISL_518623	0	28878-29902	EPI_ISL_471263
EPI_ISL_464140	0	0-23928	EPI_ISL_421655	2	23928-29902	EPI_ISL_423851
EPI_ISL_519756	2	0-28878	EPI_ISL_519506	0	28878-29902	EPI_ISL_438065
EPI_ISL_521351	2	0-28878	EPI_ISL_519476	0	28878-29902	EPI_ISL_438065
EPI_ISL_479518	1	0-24075	EPI_ISL_461481	2	24075-29902	EPI_ISL_459379
EPI_ISL_521825	2	0-28878	EPI_ISL_522065	0	28878-29902	EPI_ISL_518855
EPI_ISL_518970	2	0-28878	EPI_ISL_519303	0	28878-29902	EPI_ISL_499667
EPI_ISL_512950	0	0-3095	EPI_ISL_437716	3	3095-29902	EPI_ISL_437486
EPI_ISL_520655	2	0-28878	EPI_ISL_521787	0	28878-29902	EPI_ISL_499667
EPI_ISL_516778	3	0-25563	EPI_ISL_513293	2	25563-29902	EPI_ISL_521074
EPI_ISL_517938	3	0-27877	EPI_ISL_514211	2	27877-29902	EPI_ISL_425361
EPI_ISL_520605	2	0-28878	EPI_ISL_520165	0	28878-29902	EPI_ISL_499667
EPI_ISL_496298	0	0-28310	EPI_ISL_461482	2	28310-29902	EPI_ISL_447710
EPI_ISL_474976	3	0-27877	EPI_ISL_436059	2	27877-29424	EPI_ISL_500061
EPI_ISL_496254	3	0-24047	EPI_ISL_449979	0	24047-29902	EPI_ISL_415621
EPI_ISL_470879	3	0-9724	EPI_ISL_460103	0	9724-29902	EPI_ISL_447561
EPI_ISL_519335	2	0-28878	EPI_ISL_521589	0	28878-29902	EPI_ISL_479960
EPI_ISL_455354	0	0-8916	EPI_ISL_455352	1	8916-29902	EPI_ISL_414443
EPI_ISL_512062	3	0-28878	EPI_ISL_447033	2	28878-29902	EPI_ISL_462369
EPI_ISL_520553	2	0-28878	EPI_ISL_521067	0	28878-29902	EPI_ISL_471263
EPI_ISL_519186	2	0-28878	EPI_ISL_519279	0	28878-29902	EPI_ISL_499667
EPI_ISL_516769	3	0-27877	EPI_ISL_418036	2	27877-29902	EPI_ISL_494082
EPI_ISL_426890	3	0-27877	EPI_ISL_417830	2	27877-29902	EPI_ISL_426356
EPI_ISL_483142	0	0-4389	EPI_ISL_416578	1	4389-29902	EPI_ISL_419767
EPI_ISL_513052	0	0-3095	EPI_ISL_475666	3	3095-29902	EPI_ISL_513184
EPI_ISL_521728	2	0-28878	EPI_ISL_521796	0	28878-29902	EPI_ISL_479960
EPI_ISL_520644	2	0-28878	EPI_ISL_521036	0	28878-29902	EPI_ISL_479960
EPI_ISL_495724	2	0-28881	EPI_ISL_495647	0	28881-29902	EPI_ISL_514665
EPI_ISL_483136	3	0-27877	EPI_ISL_498042	2	27877-29902	EPI_ISL_472195
EPI_ISL_519785	2	0-28878	EPI_ISL_521813	0	28878-29902	EPI_ISL_438065

EPI_ISL_467431	2	0-13730	EPI_ISL_475093	0	13730-29902	EPI_ISL_419791
EPI_ISL_519970	2	0-28878	EPI_ISL_519312	0	28878-29902	EPI_ISL_438065
EPI_ISL_521794	2	0-28878	EPI_ISL_522240	0	28878-29902	EPI_ISL_479960
EPI_ISL_519895	2	0-28878	EPI_ISL_522065	0	28878-29902	EPI_ISL_499667
EPI_ISL_513211	0	0-3095	EPI_ISL_513162	3	3095-29902	EPI_ISL_513171
EPI_ISL_517907	3	0-27877	EPI_ISL_513293	2	27877-29902	EPI_ISL_466545
EPI_ISL_417420	3	0-20270	EPI_ISL_420307	0	20270-29902	EPI_ISL_416405
EPI_ISL_430339	1	0-20134	EPI_ISL_427772	0	20134-29902	EPI_ISL_482320
EPI_ISL_475595	0	0-14804	EPI_ISL_426680	3	14804-29902	EPI_ISL_508883
EPI_ISL_519129	2	0-28878	EPI_ISL_520891	0	28878-29902	EPI_ISL_499667
EPI_ISL_462753	0	0-4389	EPI_ISL_422816	2	4389-29902	EPI_ISL_450255
EPI_ISL_512937	3	0-27877	EPI_ISL_455778	2	27877-29902	EPI_ISL_431909
EPI_ISL_516777	3	0-25563	EPI_ISL_513293	2	25563-29902	EPI_ISL_521074
EPI_ISL_525425	2	0-18486	EPI_ISL_445270	0	18486-29902	EPI_ISL_443222
EPI_ISL_454425	1	0-26144	EPI_ISL_460084	0	26144-29902	EPI_ISL_413855
EPI_ISL_423475	3	0-27877	EPI_ISL_418377	2	27877-29902	EPI_ISL_438074
EPI_ISL_513039	3	0-27877	EPI_ISL_479854	2	27877-29902	EPI_ISL_443672
EPI_ISL_513172	3	0-20270	EPI_ISL_513148	0	20270-29902	EPI_ISL_440724
EPI_ISL_512943	0	0-3095	EPI_ISL_437299	3	3095-29902	EPI_ISL_512918
EPI_ISL_516767	3	0-27877	EPI_ISL_513293	2	27877-29902	EPI_ISL_511415
EPI_ISL_459916	0	0-25562	EPI_ISL_419761	2	25562-29902	EPI_ISL_428920
EPI_ISL_426883	3	0-27877	EPI_ISL_525541	2	27877-29902	EPI_ISL_513512
EPI_ISL_494085	3	0-7600	EPI_ISL_456033	2	7600-29902	EPI_ISL_474046
EPI_ISL_514281	1	0-28880	EPI_ISL_475682	2	28880-29902	EPI_ISL_510722
EPI_ISL_519165	2	0-28878	EPI_ISL_519312	0	28878-29902	EPI_ISL_438065
EPI_ISL_447591	0	0-14804	EPI_ISL_482575	2	14804-29902	EPI_ISL_488199
EPI_ISL_513190	3	0-23185	EPI_ISL_513163	0	23185-29902	EPI_ISL_513033
EPI_ISL_518372	2	0-28878	EPI_ISL_519303	0	28878-29902	EPI_ISL_499667
EPI_ISL_515358	3	0-17470	EPI_ISL_515319	0	17470-29902	EPI_ISL_482471
EPI_ISL_521595	2	0-28878	EPI_ISL_522225	0	28878-29902	EPI_ISL_479960
EPI_ISL_525618	0	0-14804	EPI_ISL_418159	3	14804-29902	EPI_ISL_429266
EPI_ISL_483857	3	0-24047	EPI_ISL_469047	0	24047-29902	EPI_ISL_434171
EPI_ISL_509704	0	0-3095	EPI_ISL_437299	3	3095-29902	EPI_ISL_436635
EPI_ISL_521397	2	0-28878	EPI_ISL_518123	0	28878-29902	EPI_ISL_499667
EPI_ISL_521620	2	0-28878	EPI_ISL_522101	0	28878-29902	EPI_ISL_479960
EPI_ISL_446166	0	0-14723	EPI_ISL_426680	1	14723-29902	EPI_ISL_422018
EPI_ISL_437202	0	0-3095	EPI_ISL_421911	3	3095-29902	EPI_ISL_475921
EPI_ISL_513000	0	0-3095	EPI_ISL_437199	3	3095-29902	EPI_ISL_437750
EPI_ISL_512956	3	0-27877	EPI_ISL_444276	2	27877-29902	EPI_ISL_417829
EPI_ISL_456315	3	0-18736	EPI_ISL_428388	0	18736-29902	EPI_ISL_417106
EPI_ISL_521623	2	0-28878	EPI_ISL_522253	0	28878-29902	EPI_ISL_518855
EPI_ISL_447688	0	0-3095	EPI_ISL_509695	3	3095-29902	EPI_ISL_450035
EPI_ISL_454588	0	0-4389	EPI_ISL_450235	1	4389-29902	EPI_ISL_454583
EPI_ISL_521682	2	0-28878	EPI_ISL_522238	0	28878-29902	EPI_ISL_499667
EPI_ISL_520604	2	0-28878	EPI_ISL_521836	0	28878-29902	EPI_ISL_471263
EPI_ISL_453710	0	0-4389	EPI_ISL_455626	2	4389-29902	EPI_ISL_453736
EPI_ISL_511512	2	0-28878	EPI_ISL_420400	0	28878-29902	EPI_ISL_467132
EPI_ISL_487362	3	0-27877	EPI_ISL_466638	2	27877-29902	EPI_ISL_475167
EPI_ISL_435666	0	0-3095	EPI_ISL_455352	3	3095-29902	EPI_ISL_424855
EPI_ISL_513021	0	0-23928	EPI_ISL_512995	2	23928-29902	EPI_ISL_426892

EPI_ISL_487337	1	0-28835	EPI_ISL_467479	2	28835-29902	EPI_ISL_420719
EPI_ISL_521598	2	0-28878	EPI_ISL_520179	0	28878-29902	EPI_ISL_479960
EPI_ISL_451967	1	0-20134	EPI_ISL_467148	0	20134-29902	EPI_ISL_416353
EPI_ISL_471158	3	0-27877	EPI_ISL_476814	2	27877-29902	EPI_ISL_436293
EPI_ISL_521759	2	0-28878	EPI_ISL_521452	0	28878-29902	EPI_ISL_518855
EPI_ISL_445110	0	0-12477	EPI_ISL_415743	3	12477-29902	EPI_ISL_415481
EPI_ISL_497312	2	0-27045	EPI_ISL_463454	3	27045-29902	EPI_ISL_524078
EPI_ISL_495049	3	0-28878	EPI_ISL_476873	2	28878-29902	EPI_ISL_465255
EPI_ISL_481148	0	0-12477	EPI_ISL_421911	3	12477-29902	EPI_ISL_437452
EPI_ISL_487455	1	0-20134	EPI_ISL_420368	0	20134-29902	EPI_ISL_417313
EPI_ISL_520595	2	0-28878	EPI_ISL_521589	0	28878-29902	EPI_ISL_479960
EPI_ISL_519162	2	0-28878	EPI_ISL_521777	0	28878-29902	EPI_ISL_438065
EPI_ISL_437298	0	0-3095	EPI_ISL_509700	3	3095-29902	EPI_ISL_475921
EPI_ISL_447679	0	0-14804	EPI_ISL_426680	2	14804-29902	EPI_ISL_447749
EPI_ISL_513173	0	0-8916	EPI_ISL_475584	3	8916-29902	EPI_ISL_437486
EPI_ISL_449266	0	0-4389	EPI_ISL_499625	1	4389-29902	EPI_ISL_433110
EPI_ISL_435662	0	0-14804	EPI_ISL_429104	3	14804-29902	EPI_ISL_460366
EPI_ISL_493085	0	0-3095	EPI_ISL_447568	3	3095-29902	EPI_ISL_426556
EPI_ISL_475584	0	0-23928	EPI_ISL_429084	2	23928-29902	EPI_ISL_416519
EPI_ISL_452334	0	0-23928	EPI_ISL_482575	2	23928-29902	EPI_ISL_454023
EPI_ISL_455352	0	0-8916	EPI_ISL_452761	1	8916-29902	EPI_ISL_420551
EPI_ISL_495041	3	0-27877	EPI_ISL_476858	2	27877-29902	EPI_ISL_465859
EPI_ISL_455773	0	0-14804	EPI_ISL_482575	3	14804-29902	EPI_ISL_513233
EPI_ISL_521323	2	0-28878	EPI_ISL_521731	0	28878-29902	EPI_ISL_438065
EPI_ISL_485810	1	0-20134	EPI_ISL_478003	0	20134-29902	EPI_ISL_439941
EPI_ISL_513012	0	0-23928	EPI_ISL_512995	2	23928-29902	EPI_ISL_507037
EPI_ISL_514306	3	0-28851	EPI_ISL_514284	2	28851-29424	EPI_ISL_463452
EPI_ISL_513232	0	0-8916	EPI_ISL_475584	3	8916-29902	EPI_ISL_437748
EPI_ISL_475589	0	0-14804	EPI_ISL_482575	3	14804-29902	EPI_ISL_424975
EPI_ISL_457320	0	0-27383	EPI_ISL_425845	2	27383-29902	EPI_ISL_500101
EPI_ISL_513197	0	0-3095	EPI_ISL_513178	3	3095-29902	EPI_ISL_430819
EPI_ISL_435073	0	0-3095	EPI_ISL_451352	3	3095-29902	EPI_ISL_437326
EPI_ISL_512931	0	0-3095	EPI_ISL_509700	3	3095-29902	EPI_ISL_513233
EPI_ISL_508096	1	0-18736	EPI_ISL_510298	0	18736-29902	EPI_ISL_463509
EPI_ISL_513182	0	0-3095	EPI_ISL_437199	3	3095-29902	EPI_ISL_454802
EPI_ISL_519256	2	0-28878	EPI_ISL_518473	0	28878-29902	EPI_ISL_479960
EPI_ISL_437200	0	0-3095	EPI_ISL_509695	3	3095-29902	EPI_ISL_475921
EPI_ISL_449271	0	0-4389	EPI_ISL_451318	1	4389-29902	EPI_ISL_420152
EPI_ISL_516754	3	0-25433	EPI_ISL_514177	1	25433-29902	EPI_ISL_437751
EPI_ISL_513168	0	0-3095	EPI_ISL_437745	3	3095-29902	EPI_ISL_444472
EPI_ISL_437745	0	0-3095	EPI_ISL_452334	3	3095-29902	EPI_ISL_437483
EPI_ISL_454592	2	0-15438	EPI_ISL_416140	1	15438-29902	EPI_ISL_450198
EPI_ISL_508044	3	0-27384	EPI_ISL_512795	2	27384-29902	EPI_ISL_511535
EPI_ISL_437203	0	0-14804	EPI_ISL_426680	1	14804-29902	EPI_ISL_516988
EPI_ISL_449261	0	0-4389	EPI_ISL_451513	1	4389-29902	EPI_ISL_426009
EPI_ISL_483871	3	0-27877	EPI_ISL_476858	0	27877-29902	EPI_ISL_428893
EPI_ISL_513046	0	0-23928	EPI_ISL_512995	2	23928-29902	EPI_ISL_487933
EPI_ISL_463669	0	0-14804	EPI_ISL_482575	2	14804-29902	EPI_ISL_428895
EPI_ISL_521621	2	0-28878	EPI_ISL_520175	0	28878-29902	EPI_ISL_518855
EPI_ISL_519626	2	0-28878	EPI_ISL_518963	0	28878-29902	EPI_ISL_479960

EPI_ISL_467625	0	0-8916	EPI_ISL_493179	3	8916-29902	EPI_ISL_458020
EPI_ISL_518935	2	0-28878	EPI_ISL_519266	0	28878-29902	EPI_ISL_441368
EPI_ISL_519468	2	0-28878	EPI_ISL_480641	0	28878-29902	EPI_ISL_499667
EPI_ISL_483706	3	0-11109	EPI_ISL_444586	0	11109-29902	EPI_ISL_416629
EPI_ISL_521572	2	0-28878	EPI_ISL_521853	0	28878-29902	EPI_ISL_479960
EPI_ISL_516779	3	0-27877	EPI_ISL_419544	2	27877-29902	EPI_ISL_445315
EPI_ISL_508020	3	0-27384	EPI_ISL_508873	2	27384-29902	EPI_ISL_480154
EPI_ISL_497082	3	0-27877	EPI_ISL_495771	2	27877-29552	EPI_ISL_458466
EPI_ISL_420343	3	0-27877	EPI_ISL_429968	2	27877-29902	EPI_ISL_437998
EPI_ISL_522541	3	0-24047	EPI_ISL_510588	0	24047-29902	EPI_ISL_427813
EPI_ISL_509874	1	0-19838	EPI_ISL_448101	2	19838-29902	EPI_ISL_458229
EPI_ISL_521846	2	0-28878	EPI_ISL_480641	0	28878-29902	EPI_ISL_518855
EPI_ISL_493070	1	0-20134	EPI_ISL_439344	0	20134-29902	EPI_ISL_422786
EPI_ISL_507011	3	0-27877	EPI_ISL_416753	2	27877-29902	EPI_ISL_464231
EPI_ISL_519455	2	0-28878	EPI_ISL_480641	0	28878-29902	EPI_ISL_471263
EPI_ISL_521324	2	0-28878	EPI_ISL_520526	0	28878-29902	EPI_ISL_438065
EPI_ISL_476267	0	0-4389	EPI_ISL_500401	2	4389-29902	EPI_ISL_515541
EPI_ISL_449259	0	0-4389	EPI_ISL_415609	2	4389-29902	EPI_ISL_439243
EPI_ISL_520948	2	0-28878	EPI_ISL_520268	0	28878-29902	EPI_ISL_479960
EPI_ISL_520810	2	0-28878	EPI_ISL_522217	0	28878-29902	EPI_ISL_479960
EPI_ISL_478469	3	0-27877	EPI_ISL_481739	2	27877-29902	EPI_ISL_421783
EPI_ISL_439137	0	0-8916	EPI_ISL_488783	1	8916-29902	EPI_ISL_439093
EPI_ISL_518936	2	0-28878	EPI_ISL_519052	0	28878-29902	EPI_ISL_441368
EPI_ISL_520231	2	0-28878	EPI_ISL_519294	0	28878-29902	EPI_ISL_499667
EPI_ISL_437199	0	0-3095	EPI_ISL_509695	3	3095-29902	EPI_ISL_475921
EPI_ISL_521429	2	0-28878	EPI_ISL_518927	0	28878-29902	EPI_ISL_499667
EPI_ISL_440589	3	0-27877	EPI_ISL_508873	2	27877-29902	EPI_ISL_492160
EPI_ISL_476327	3	0-25563	EPI_ISL_444534	2	25563-29902	EPI_ISL_470615
EPI_ISL_451185	0	0-14804	EPI_ISL_482575	2	14804-29902	EPI_ISL_437090
EPI_ISL_518513	2	0-28878	EPI_ISL_519046	0	28878-29902	EPI_ISL_499667
EPI_ISL_437299	0	0-3095	EPI_ISL_513182	3	3095-29902	EPI_ISL_475921
EPI_ISL_519175	2	0-28878	EPI_ISL_519657	0	28878-29902	EPI_ISL_499667
EPI_ISL_519782	2	0-28878	EPI_ISL_519506	0	28878-29902	EPI_ISL_438065
EPI_ISL_513162	0	0-3095	EPI_ISL_447669	3	3095-29902	EPI_ISL_512934
EPI_ISL_495226	2	0-18486	EPI_ISL_471929	0	18486-29902	EPI_ISL_454991
EPI_ISL_495051	3	0-27877	EPI_ISL_475033	2	27877-29902	EPI_ISL_465859
EPI_ISL_513007	1	0-20134	EPI_ISL_417999	0	20134-29902	EPI_ISL_437472
EPI_ISL_519576	2	0-28878	EPI_ISL_522228	0	28878-29902	EPI_ISL_479960
EPI_ISL_519550	2	0-28878	EPI_ISL_519034	0	28878-29902	EPI_ISL_438065
EPI_ISL_520515	2	0-28878	EPI_ISL_520316	0	28878-29902	EPI_ISL_518855
EPI_ISL_524226	0	0-3095	EPI_ISL_486105	3	3095-29902	EPI_ISL_524368
EPI_ISL_441321	3	0-27877	EPI_ISL_456055	2	27877-29902	EPI_ISL_457804
EPI_ISL_464547	0	0-27383	EPI_ISL_464180	2	27383-29902	EPI_ISL_455854
EPI_ISL_420451	1	0-28835	EPI_ISL_420449	2	28835-29902	EPI_ISL_454454
EPI_ISL_512995	0	0-23928	EPI_ISL_447669	2	23928-29902	EPI_ISL_507037
EPI_ISL_452934	3	0-27877	EPI_ISL_417336	2	27877-29902	EPI_ISL_421199

Supplementary Table 4. *List of SARS-CoV-2 genomes flagged by Bolotie as recombinant. For each unique GISAID identifier listed in column 1, we show its parental clades, approximate breakpoint position, and closest parents for each of the parental clades.*

References

Hadfield, J., Megill, C., Bell, S. M., Huddleston, J., Potter, B., Callender, C., Sagulenko, P., Bedford, T., & Neher, R. A. (2018). Nextstrain: real-time tracking of pathogen evolution. *Bioinformatics*, *34*(23), 4121–4123.

Shu, Y., & McCauley, J. (2017). GISAID: Global initiative on sharing all influenza data–from vision to reality. *Eurosurveillance*, *22*(13), 30494.

VanInsberghe, D., Neish, A. S., Lowen, A. C., & Koelle, K. (2020). Identification of SARS-CoV-2 recombinant genomes. *BioRxiv*.
